# Human Organoids for Rapid Validation of Gene Variants Linked to Cochlear Malformations

**DOI:** 10.21203/rs.3.rs-4474071/v1

**Published:** 2024-06-11

**Authors:** Mohammad Faraz Zafeer, Memoona Ramzan, Duygu Duman, Ahmet Mutlu, Serhat Seyhan, Tayyar Kalcioglu, Suat Fitoz, Brooke A. DeRosa, Shengru Guo, Derek M. Dykxhoorn, Mustafa Tekin

**Affiliations:** University of Miami Miller School of Medicine; University of Miami Miller School of Medicine; Ankara University Faculty of Health Sciences; Istanbul Medeniyet University; Memorial Şişli Hospital; Istanbul Medeniyet University; Ankara University School of Medicine; University of Miami Miller School of Medicine; University of Miami Miller School of Medicine; University of Miami Miller School of Medicine; University of Miami Miller School of Medicine

**Keywords:** Candidate gene, Cochlear malformation, CRISPR/Cas9, Deafness, Inner ear anomaly, iPSC, Organoids, Variants of uncertain significance

## Abstract

Developmental anomalies of the hearing organ, the cochlea, are diagnosed in approximately one-fourth of individuals with congenital deafness. Most patients with cochlear malformations remain etiologically undiagnosed due to insufficient knowledge about underlying genes or the inability to make conclusive interpretations of identified genetic variants. We used exome sequencing for genetic evaluation of hearing loss associated with cochlear malformations in three probands from unrelated families. We subsequently generated monoclonal induced pluripotent stem cell (iPSC) lines, bearing patient-specific knockins and knockouts using CRISPR/Cas9 to assess pathogenicity of candidate variants. We detected *FGF3* (p.Arg165Gly) and *GREB1L* (p.Cys186Arg), variants of uncertain significance in two recognized genes for deafness, and *PBXIP1*(p.Trp574*) in a candidate gene. Upon differentiation of iPSCs towards inner ear organoids, we observed significant developmental aberrations in knockout lines compared to their isogenic controls. Patient-specific single nucleotide variants (SNVs) showed similar abnormalities as the knockout lines, functionally supporting their causality in the observed phenotype. Therefore, we present human inner ear organoids as a tool to rapidly validate the pathogenicity of DNA variants associated with cochlear malformations.

## Introduction

Approximately one in 500 newborns are diagnosed with permanent hearing loss (HL) (Bainbridge & Wallhagen 2014; Petit et al. 2023). Inner ear anomalies (IEAs) affecting the cochlea are reported in about 25% of these children (Bamiou et al. 2000; Brotto et al. 2021; Ocak et al. 2019). Studies performed in various animal models shed light on fundamental mechanisms governing vertebrate inner ear development (Chen et al. 2017; Torii et al. 2016; Whitfield et al. 2002). However, mutations in relatively few of the genes recognized in animal model systems have been shown to cause cochlear malformations in humans.

Interpretation of the identified variants through genetic testing in HL requires collating and analyzing the available literature for supporting evidence, followed by a formal classification based on this evidence (Patel et al. 2021). A recent study shows that 70% of the identified missense variants in people with HL are classified as variants of uncertain significance (VUS) (Tollefson et al. 2023). Functional studies can help establish causality in these cases, which are often lacked in published studies.

It is difficult to directly examine the molecular and cellular processes leading to the establishment of the human inner ear. It is located deep within the skull surrounded by bone and other tissues (Nomura et al. 2014). There is also a paucity of biopsy material appropriate for molecular analysis, particularly in the early stages of development. Finally, the range of testing that can be carried out on human embryos and fetuses is limited by ethical considerations, further complicating research (Plomer 2013).

The recent advancements in stem cell technology have enabled us to create three-dimensional organoids similar to developing human ears using human induced pluripotent stem cells (iPSCs) (Doda et al. 2023; Koehler et al. 2017; Qi et al. 2024; Steinhart et al. 2022; van der Valk et al. 2023). These iPSC-derived structures imitate the basic cartography of the developing ear and can, therefore, be used to investigate how development at the cellular stage happens in this organ (Doda et al. 2023; Koehler et al. 2017; Romano et al. 2022; Steinhart et al. 2022; van der Valk et al. 2023). Generating inner ear organoids (IEOs) involves differentiating pluripotent stem cells into otic placode-like cells. These cells later form cavities resembling otic vesicles that contain hair cells, supporting cells, and neurons like those seen in the inner ear after maturation (Koehler et al. 2017; Moore et al. 2023; Nie & Hashino 2020; Romano et al. 2022; Tang et al. 2020). Organoids hold several advantages over animal models, including the ability to study human-specific aspects of inner ear development and disease with much shorter timelines than animal models (Qi et al. 2024). These lines retain the genetic architecture of the cells from whom the lines were derived and are amenable to genomic engineering approaches, including CRISPR/Cas9-based methods (Rivron et al. 2023).

In this study, we investigate the impact of DNA variants in known cochlear malformation genes *FGF3* (Fibroblast Growth Factor 3) and *GREB1L* (GREB1 Like Retinoic Acid Receptor Coactivator), along with a candidate gene, *PBXIP1* (PBX Homeobox Interacting Protein 1), in inner ear development via IEOs. *FGF3* participates in the early development of the inner ear (Tekin et al. 2007). Pathogenic variants in *FGF3* cause deafness with LAMM (Labyrinthine Aplasia, Microtia, and Microphthalmia; OMIM 610706), an autosomal recessively-inherited syndrome characterized by missing inner ear structures as well as small external ears and teeth. *GREB1L* has a role in neural crest development and retinoic acid pathway (Brophy et al. 2017; Vega-Lopez et al. 2018). Several reports have associated *GREB1L* variants with autosomal dominant urogenital or cochlear anomalies (Adadey et al. 2022; De Tomasi et al. 2017; Herlin et al. 2019; Jacquinet et al. 2020; Kim et al. 2022; Schrauwen et al. 2018a). In two patients with cochlear malformations, we detected previously unreported missense variants in *FGF3* and *GREB1L*, interpreted as VUS. In another patient, we detected a nonsense variant in *PBXIP1*, a gene not previously associated with a human phenotype. By establishing monoclonal IEOs for knockout and patient-specific variants of these genes, we show differences in organoid size, number of luminal spaces (otic vesicles), and lower expression of otic vesicle markers in both knockout and variant-bearing organoids compared to controls.

## Methods

### Enrollment of subjects and exome sequencing of probands

The study was approved by the Institutional Review Board (Protocol no. 20081138) at the University of Miami (USA) and Ethics Committee (Protocol no. 012413) at Ankara University Medical School (Türkiye). Written informed consents were obtained from all participants and in the case of minors, it was obtained from parents. Audiometry was performed to measure average hearing thresholds for all participants under standard conditions and guidelines. All affected individuals were examined by a clinical geneticist and otolaryngologist.

For exome sequencing (ES), we followed a recently published protocol (Ramzan et al. 2024). Briefly, single nucleotide, indel, and copy number variants (CNVs) in all known deafness genes were analyzed. Variants were retained for further evaluation if they had an allele frequency of less than 0.01. The variants in all known genes for HL were analyzed using a larger list retrieved from hereditary hearing loss homepage (https://hereditaryhearingloss.org/) and OMIM. If there was no candidate variant identified in known deafness genes, ES data were re-examined for all genes containing variants less than 0.01 allele frequency. CNV analysis with ES data used CoNIFER v.02.2 with default parameters. It uses a singular value decomposition method to correct systematic biases and identifies a CNV call if the corrected signal reaches a predefined threshold at no less than three consecutive exons (Krumm et al. 2012). We performed Sanger sequencing to confirm candidate variants and evaluate segregation within respective families.

Candidate variants were classified according to ACMG and ClinGen Hearing Loss Expert Panel (HL-EP) specifications, aligned with the ACMG/AMP Variant Interpretation Guidelines (Oza et al. 2018; Richards et al. 2015; Tavtigian et al. 2020). AlphaMissense was included in the prediction of pathogenicity as described (Zhuo et al. 2024).

### iPSC maintenance and validation

Cells from a validated male European lymphocyte-derived iPSC line (ASE9203) were cultured on Vitronectin (Thermo Scientific, USA, Cat# A2858501) coated culture ware and maintained in E8-Flex (Thermo Scientific, USA, Cat# A2858501) media supplemented with 100 μg/mL Normocin (Invivogen, USA, Cat# ant-nr-05), following the manufacturer’s guidelines. Routine passages utilized 1x Revitacell (Thermo Scientific, USA, Cat# A2644501). We regularly assessed pluripotency markers OCT4 (Cell Signaling Technology, USA, Cat# 2840) and TRA-1–60 (Cell Signaling Technology, USA, Cat# 4746) through Immunocytochemistry (ICC).

### Generation of CRISPR/Cas9-edited variants

We generated monoclonal cell lines for *FGF3*^*KO*^, *FGF3*^*c.493A>G*^, *GREB1L*^*KO*^, *GREB1L* ^*c.556T>C*^, and *PBXIP1*^*c.1722G>A*^ as previously described (Ramzan et al. 2024). Custom sgRNA and HDR donor blocks were designed and procured using the Alt-R-CRISPR/Cas9 platform (IDT); sequences are available in supplementary Table 1. Cas9-gRNA ribonucleoprotein complexes were assembled under sterile conditions using 140 pmol sgRNA and 40 pmol EnGen^®^ Spy Cas9 HF1 (New England Biolabs, USA, Cat#M0667M) and incubated for 10 mins at room temperature. Following the incubation, 8×10^5^ cells were suspended in the RNP complex and premixed 3 μM HDR donor block in 80 μL of nucleofector solution. The final volume of the nucleofection mix was 100 μL. Nucleofections are performed using the P3-primary Cell 4D-Nucleofector Kit (Lonza, USA, Cat# V4XP-3024) and preprogrammed pulse protocol CA-137 in the 4D-Nucleofector system (Lonza). Post-nucleofections, iPSCs were cultured in E8-flex media supplemented with CloneR^™^2 (Stem Cell Technologies, Canada, Cat# 100–0691) and 1 μM of the HDR enhancer-V2 (IDT).

### DNA isolation and confirmation of CRISPR/Cas9-edited cell pools and off-targets

DNA isolation was done using QuickExtract DNA Extraction Solution (Biosearch Technologies, USA, Cat# QE09050); the resulting DNA was then PCR amplified using Phusion^®^ High-Fidelity PCR Master Mix (New England Biolabs, USA, Cat# M0531S) and sequenced with primers flanking (Supplementary Table S1) the guide region and Sanger sequencing at Genewiz (Azenta Life Sciences). Outcomes of CRISPR/Cas9 knock-ins were assessed using ICE Analysis (Conant et al. 2022). For every guide sequence, off-target analysis was done using bioinformatic tools such as Cas-OFFinder (Bae et al. 2014) and the sgRNA guide analysis tool from IDT in Coralville, Iowa, USA. The top five off-target sites were Sanger sequenced for flanking primers and analyzed for all the clonal lines used in this study (Supplementary Table S2).

### Isolation of monoclonal lines

Single-cell monoclonal lines were isolated using Poisson distribution (Sanjurjo-Soriano et al. 2022). CRISPR/Cas9 pools were briefly dissociated using StemPro accutase (Thermo Scientific, USA, Cat# A1110501). The resulting cell suspension was collected in E8-Flex media (Thermo Scientific, USA, Cat# A2858501) containing CloneR^™^2 (Stem Cell Technologies, Canada, Cat# 100–0691) and centrifuged at 300 rpm. After centrifugation, the single-cell suspension was passed through 70 μm strainers (SP Bel-Art, USA, Cat# H13680–0070), and the cells were then counted using a Countess II cell counter (Thermo Scientific, USA). Depending on the cell concentrations, serial dilutions were performed to achieve a typical concentration of 1 cell per 100 μL of media. The cells were then plated in 96-well plates precoated with rhLaminin (Thermo Scientific, USA, Cat# A29249), and media changes were done with 1x CloneR^™^2-containing E8 media every other day for the first week and then with 0.5x CloneR^™^2 containing E8-Flex media till the single cell colonies reached a passaging confluence. Clonal lines were identified using Sanger sequencing, and from the same experiments, clonal lines bearing large frameshift deletions disrupting the open reading frame were used as knockouts for *FGF3* and *GREB1L* (Supplementary Figure S1).

### Generation of inner ear organoids

IEOs were generated using the previously described protocol by (Moore et al. 2023). Briefly, on the first day of differentiation (day – 2), cells were dissociated with StemPro accutase (Thermo Scientific, USA, A1110501). The cells were then passed through 70 μm strainers (SP Bel-Art, USA, Cat# H13680–0070), and 3500 cells/well were plated in 96-well Nunc-Sphera U-bottom plates (Thermo Scientific, USA, Cat# 174925) in 100 μL of E8-Flex media containing 20 μM Y-27632 (Stem Cell Technologies, Canada, Cat# 72304). After 4 hours of initial plating, 100 μL of fresh E8-Flex media was added to each well, bringing the final concentration of Y-27632 to 10 μM. All the aggregates were collected on day 0, washed thrice with E6-Medium (Thermo Scientific, USA, Cat# A1516401) and IEO differentiation media (E6-Medium, 2% GFR-Matrigel (Corning, Sigma Aldrich, Cat# 354230) 10 μM SB431542 (Stem Cell Technologies, Canada, Cat# 72232), 4 ng/mL FGF2 (Peprotech, Thermo, USA, Cat# 100–18B) and 2.5 ng/mL BMP4 (Stemgent, USA, Cat# 03–0007)). Cell aggregates were transferred to a new 96-well Nunc-Sphera U-bottom plate in 100 μL of differentiation media. On day 3, 25 μL E6-Medium containing 100 μg/mL Normocin, 250 ng/mL FGF-2, and 200 nM LDN193189 (Stem Cell Technologies, Canada, Cat# 72147) were added, bringing the volume to 125 μL. On days 6 and 9 of differentiation, cell aggregates were washed thrice with E6 media and then thrice with E6 media containing 100 μg/mL Normocin, 3 mM CHIR99021 (Stemgent, USA, Cat# 04–0004-02), 200 nM LDN193189, and 50 ng/mL FGF2. The cell aggregates were transferred to fresh 96- well U-bottom plates in 250 μL of new media.

Cell aggregates were washed thrice on the 11th day of differentiation with Advanced DMEM/F12 (Thermo Scientific, USA, Cat# 12634010). Then they were transferred to 90 mm Nunc low-attachment plates (Thermo Scientific, USA, Cat# 174945) in 10 mL of organoid maturation media (OMM) comprising Advanced DMEM/F12, Neurobasal medium (Thermo Scientific, USA, Cat# 21103049), 1X Glutamax, 0.5X B-27 supplement without Vitamin A, 0.5X N-2 supplement, 0.1 mM 2-Mercaptoethanol, 100 μg/mL Normocin, and supplemented with 1% GFR-Matrigel and 3 μM CHIR99021. On days 13 and 15, culture media was changed to OMM + 3 mM CHIR99021 + 1 mM Purmorphamine (Stem Cell Technologies, Canada, Cat# 72202). On day 18, aggregates were washed to eliminate CHIR99021, and the media was changed to OMM + 3 mM IWP-2 (Stem Cell Technologies, Canada, Cat# 72122) + 1 mM Purmorphamine. The media was changed on day 20, with fresh media used on day 18. On day 22, cultures were washed and transferred to an anti-adhere solution coated with a low attachment 100 mm culture dish in OMM-only. The cultures received half media change every 3rd day and complete media change every 7th day until day 60. Samples were collected on day 25 and day 35 for RNA sequencing and on day 25, day 35, and day 60 for immunohistochemistry.

### Immunohistochemistry and sectioning

IEOs on day 25 and day 60 were collected, washed with PBS twice, fixed with 4% paraformaldehyde, and processed at the Cancer Modeling Shared Resource core, Sylvester Cancer Center, University of Miami. Serial sections of 5 μm thickness were antigen-retrieved using 10 mM citrate buffer pH6. For immunohistochemistry, sections were permeabilized with 0.4% triton-X for 10 mins and blocked with 5% BSA + 0.01% Tween20. Primary antibody incubations for MYO7A 1:50 ((MYO7A 138–1, deposited to the DSHB by Orten, D.J. (DSHB Hybridoma Product MYO7A 138–1)), and SOX2 1:100 (Cell Signaling Technology, USA, Cat# 3579) was done in the blocking buffer for overnight at 4°C. The following sections were washed thrice with PBS the following day, and secondary antibody incubations at 1:500 dilutions for Anti-Mouse AlexaFluor-647 (Thermo Scientific, USA, Cat# A32728TR) and anti-Rabbit AlexaFluor-488 (Thermo Scientific, USA, Cat# A32787TR) were done for 1hr at room temperature, respectively. Slides were mounted with ProLong^™^ Glass Antifade (Thermo, USA, Cat# P36980) and images were acquired using Zeiss LSM 980 with AiryScan 2 (Zeiss, Germany) at Flow Cytometry Shared Resource (FCSR), University of Miami. All the images were analyzed using the Fiji Image Analysis Tool or ImageJ (Schindelin et al. 2012).

### Western blot analysis

Cells were harvested in RIPA buffer supplemented with 1x HALT protease and phosphatase inhibitor (Thermo Scientific, USA, Cat# 78441). Protein quantification was performed using a Thermo Scientific^™^ Pierce^™^ BCA kit (Thermo Scientific, USA, Cat# 23227). Equal amounts of protein were then reduced and loaded onto a 4–20% Tris-Glycine gradient gel for separation, following the method described by Laemmli (Laemmli 1970). Subsequently, proteins were transferred onto a 0.22 μm PVDF membrane using the Turbo-trans Blot system (Biorad, USA). The membranes were then blocked in 5% BSA for 1.5 hours and incubated overnight at 4°C with primary PBXIP1 antibody (Proteintech, Thermo Scientific, USA, Cat# 12102–1-AP) diluted at 1:1000 in 5% BSA + TBST (TBS with 0.5% Tween). After washing with TBST, the blots were incubated with HRP-conjugated anti-rabbit goat secondary antibody (1:3000) diluted in 5% BSA + TBST for 1.5 hours at room temperature. Following the termination of antibody reactions, the blots were washed three times with TBST and developed using the West Pico Super-Signal ECL substrate (Thermo Scientific, USA, 37069). Finally, visualization was performed using FluorChemE (ProteinSimple, USA).

### mRNA expression analysis

The expression of *GREB1L*, *FGF3*, and *PBXIP1* mRNA in monoclonal lines was analyzed using qRT-PCR. Total RNA was isolated with TRIzol Reagent (Thermo, USA, Cat# 15596026) according to the manufacturer’s instructions. cDNA was synthesized using qScript XLT cDNA SuperMix (Quanta Biosciences, USA, Cat# 9516–025). The primers amplifying the transcript were *GREB1L* sense 5’-CAGTTTCCTGGCATCACATTTC-3’ antisense 5’-GTAACCACACTGTCTCCTCTTC-3’; FGF3 sense 5’-ATTGCTCCTGGGTGGAAATTA-3’ antisense 5’-AGAGAGAAAGAGAGGGAGAGTG-3’; *PBXIP1* sense 5’-GGCCTCTCTGCTAAGAACATAC-3’ antisense 5’-GATGCCATCCTCACCAAAGA-3’.

### Quantification and statistical analysis

All the statistical analyses are performed using GraphPad Prism 10. Paired analyses were done using the student’s t-test. Multiple comparisons were performed using one-way ANOVA with Tukey’s multiple comparisons test. The results are expressed as Mean ± SEM; a statistical difference of p ≤ 0.05 was considered significant. The significant differences are marked with (*) whenever comparisons were made between *GREB1L*^*c.556T>C*^, *FGF3c*^*.493A>G*^, *FGF3*^*KO*^, *GREB1L*^*KO*^, *PBXIP1*^*c.1722G>A*^ and their respective controls *GREB1L*^*WT*^, *FGF3*^*WT*^*, PBXIP1*^*WT*^. Details about the number of replicates and significance notation are provided in the figure legends.

## Results

### Identification of candidate genes and variants

**Figure 1:**
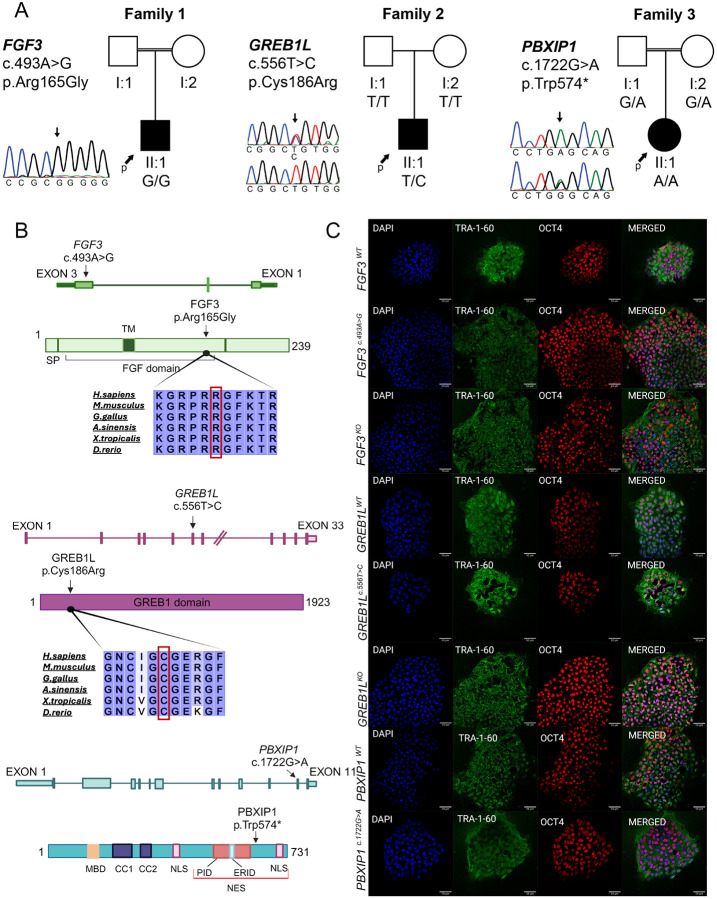
Pedigrees, variants, and confirmation of pluripotency in edited lines. **(a)** Graphical representation of participating families, segregating variants, and their location on respective gene and protein. Squares represent males, and circles indicate females. Filled symbols show affected individuals. Arrows point to the probands (p) of the respective families. The mutated residue is mentioned with an arrow in each chromatogram as well as in the protein schematic. **(b)** Graphical representation of mutated genes, proteins, and localization of variants. **(c)** Immunostaining of OCT4 and TRA-1–60 in monoclonal lines derived after CRISPR/Cas9 editing in *GREB1L*^*WT*^, *GREB1L*^*c.556T>C*^, *GREB1L*^*KO*^, *FGF3*^*WT*^, *FGF3*^*c.493A>G*^, *FGF3*^*KO*^, *PBXIP1*^*WT*^ and *PBXIP1*^*c.1722G>A*^, respectively. **SP:** Signal Peptide, **MBD:** Microtubule Binding Domain, **CC1:** Coil-coiled Domain 1, **CC2:** Coil-coiled Domain 2, **NLS:** Nuclear Localization Sequence, **PID:** PBX1 interacting Domain, **ERID:** Erα interacting Domain, **NES:** Nuclear Export Sequence.

In our ongoing studies on HL, we identified an individual homozygous for an *FGF3* variant (c.493A > G; p.Arg165Gly) and another individual who is heterozygous for a *GREB1L* variant (c.556T > C; p.Cys186Arg) (Fig. 1a–b and Supplementary Table S3). The proband with the *FGF3* variant is a 6-year-old male with bilateral congenital profound deafness whose temporal bone CT scan showed bilateral labyrinthine aplasia. Physical examination revealed normal-sized but prominent external ears and widely spaced lower incisor teeth. The parents were first cousins without HL. The proband with the GREB1L variant is a 5-year-old male with congenital profound sensorineural HL in the left ear associated with common cavity malformation (Supplementary Figure S2A). Hearing and imaging studies in the right ear are normal. An ultrasound examination for kidney and urinary system anomalies is unremarkable. His developmental history is normal. Parents have normal hearing and do not have the variant detected in the proband. Although both variants are highly conserved among different vertebrate species (Fig. 1b), following ACMG guidelines, both variants are interpreted as VUS (Supplementary Table S3). Thus, increasing the certainty of their pathogenicity depends on functional abnormalities that additional studies can demonstrate.

In the same cohort, we identified a candidate gene, *PBXIP1*, for bilateral cochlear aplasia. The proband is an 8-year-old female who was born with bilateral profound sensorineural HL without additional abnormalities. CT scans of the temporal bone showed bilateral cochlear aplasia (Supplementary Figure S2B). Initially, the search for variants in known deafness genes ended with no variant of interest that could be associated with HL in this family. Parents were consanguineous and there were 11 regions of homozygosity greater than 2Mb in the proband (Supplementary Table S4). After filtering of variants and Sanger sequencing of family members, only one variant co-segregated with the phenotype: the proband is homozygous for the nonsense variant c.1722G > A (p.Trp574*) in *PBXIP1* and parents are heterozygous (Fig. 1a and Supplementary Table S3). Variants in this gene have not been previously associated with human phenotypes. PBXIP1 is present in the nucleoplasm and cytoplasm of cells in most tissues (https://www.proteinatlas.org/); the gEAR database (https://www.umgear.org/) shows its highest expression in prosensory duct floor and lateral duct floor in developing human cochlea along with significant expression in all other parts of cochlea, such as roof, periotic mesenchymal cells, and medial duct floor (Supplementary Figures S3 and S4) (van der Valk et al. 2023).

### Generation of isogenic knockout and variant-bearing iPSCs via CRISPR/Cas9

The *GREB1L*^*c.556T>C*^, *GREB1L*^*KO*^, *FGF3* ^*c.493A>G*^*, FGF3*^*KO*^, and *PBXIP1*^*c.1722G>A*^ variants introduced into a control iPSC cell line were confirmed by Sanger sequencing (Supplementary Figure S1). Sanger sequencing of the *in silico* predicted off-target genomic loci showed no unintended mutation had been introduced into the monoclonal iPSC lines (Supplementary Table S2). All the monoclonal iPSC lines generated in the study retained their pluripotency as assessed by immunocytochemical staining for the pluripotency markers OCT4 and TRA1–60 (Fig. 1c).

### Effects of knockout and variant incorporation on gene expression

**Figure 2:**
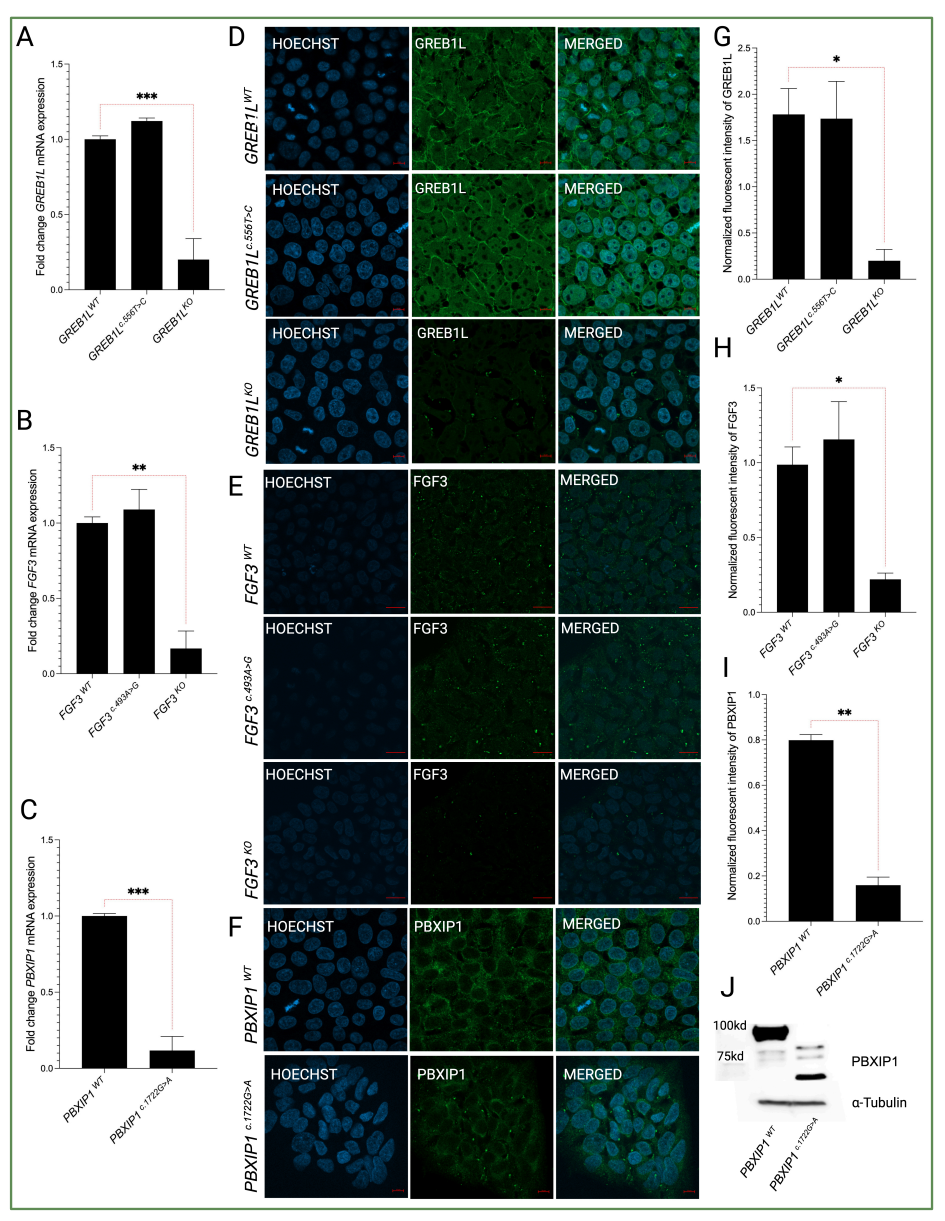
Expression of GREB1L, FGF3, and PBXIP1 in WT and edited lines. **(a)** qRT-PCR analysis of *GREB1L* expression levels in *GREB1L*^*WT*^, *GREB1L*^*c.556T>C*^, and *GREB1L*^*KO*^. **(b)** qRT-PCR analysis of *FGF3* expression levels in *FGF3*^*WT*^, *FGF3*^*c.493A>G*^ and *FGF3*^*KO*^. **(c)** qRT-PCR analysis of *PBXIP1* expression levels in *PBXIP1*^*WT*^ and *PBXIP1*^*c.1722G>A*^. **(d, g)** Effect of missense p.Cys186Arg variant on GREB1L in iPSCs. **(e, h)** Effect of missense p.Arg165Gly variant on FGF3 in iPSCs. **(f, i)** Effect of nonsense variant p.Trp574* showing slight change in signal for PBXIP1 **(j)** Western blot showing the smaller sized band in mutated PBXIP1. The results are expressed as Mean ± SEM (n=3), and the statistical difference of p ≤ 0.05 was considered significant. The significant differences are marked with (*) whenever comparisons were made between edited lines and their respective controls. *: p<0.05; **: p<0.01; ***: p<0.001.

Analysis of gene and protein levels for the missense variant-bearing monoclonal iPSC lines *GREB1L*^*c.556T>C*^ (Figs. 2a, d, and g) and *FGF3*^*c.493A>G*^ (Figs. 2b, e, and h) showed no significant differences in expression compared to their respective wild-type (WT) parental iPSC line (i.e. *GREB1L*^*WT*^ and *FGF3*^*WT*^). In contrast, the monoclonal iPSC lines bearing the CRISPR/Cas9-derived knockout of *GREB1L* (*GREB1L*^*KO*^) (Figs. 2a, d, and g) and *FGF3* (*FGF3*^*KO*^) (Figs. 2b, e, and h) showed significantly abrogated expression of these genes compared to their respective parental lines. The *PBXIP1*^*c.1722G>A*^ nonsense variant is predicted to produce a premature termination codon, potentially resulting in a truncated protein. Nonsense-mediated decay (NMD) is an mRNA quality control mechanism eukaryotic cells use to degrade mRNAs that harbor premature termination codons. The *PBXIP1*c.1722G > A variant is located 375 nucleotides away from the last intron, which makes NMD likely. A significant decrease in *PBXIP1* mRNA expression in monoclonal line bearing c.1722G > A was observed (p ≤ 0.001) consistent with the mRNA undergoing NMD (Fig. 2c). Immunofluorescent analysis of PBXIP1 showed a reduction of PBXIP1 in the mutant cells compared to WT cells (Figs. 2f and i). Immunoblot analysis of whole cell lysates from *PBXIP1*^*WT*^ and *PBXIP1*^*c.1722G>A*^ lines show that *PBXIP1*^*c.1722G>A*^ variant produced truncated protein as indicated by the detection of a smaller band of around 62.83 kilodaltons consistent with the predicted size of the truncated protein (https://www.bioinformatics.org/sms/prot_mw.html) (Fig. 2j). In addition to the production of a truncated protein, the amount of PBXIP1 protein was decreased in the PBXIP1c.1722G > A cells compared to the WT parental line. These results suggest that c.1722G > A leads to NMD with a reduced amount of truncated PBXIP1 protein being produced.

### Inner ear organoids from variant-bearing iPSCs show size reduction during development

**Figure 3:**
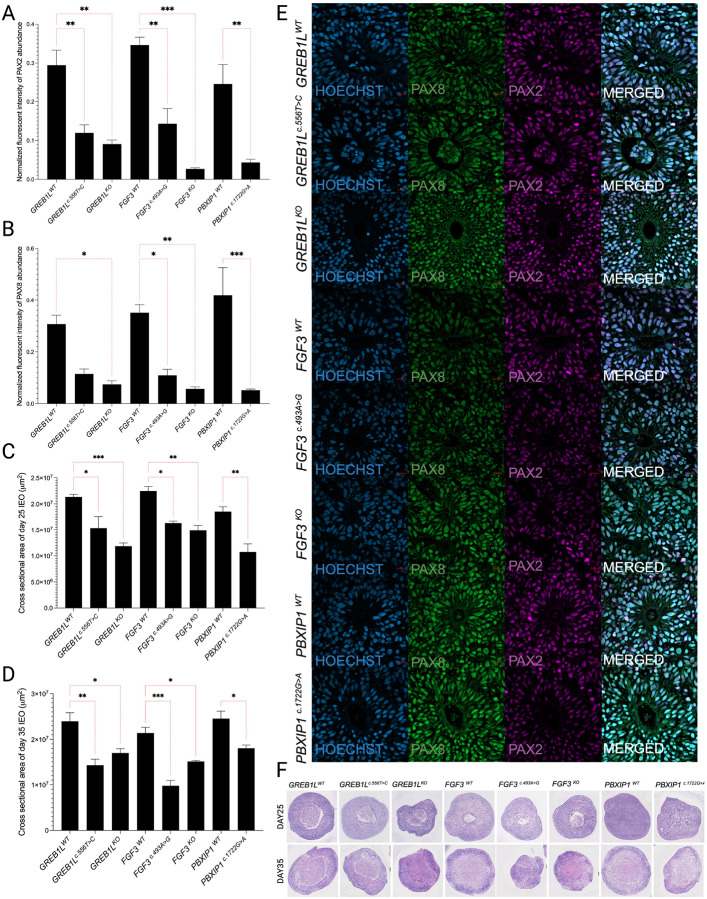
Differences of early otic lineage markers and cross-sectional area in WT and edited lines. **(a, b)** PAX2 and PAX8 quantification in inner ear organoids (IEOs) (n=3), respectively. The results are expressed as Mean ± SD, and the statistical difference of p ≤ 0.05 was considered significant. The significant differences are marked with (*) whenever comparisons were made between *GREB1L*^*c.556T>C*^, *FGF3*^*c.493A>G*^, *FGF3*^*KO*^, *GREB1L*^*KO*^, *PBXIP1*^*c.1722G>A*^, and their respective controls *GREB1L*^*WT*^, *FGF3*^*WT*^, *PBXIP1*^*WT*^. **(c, d)** Cross-sectional area analysis of day 25 and day 35 IEO (n=3), respectively. Area quantifications were done using ImageJ; results are expressed as Mean ±SD, and the statistical difference of p ≤ 0.05 was considered significant. The significant differences are marked with (*) whenever comparisons were made between *GREB1L*^*c.556T>C*^, *FGF3*^*c.493A>G*^, *FGF3*^*KO*^, *GREB1L*^*KO*^, *PBXIP1*^*c.1722G>A*^, and their respective controls *GREB1L*^*WT*^, *FGF3*^*WT*^, *PBXIP1*^*WT*^. **(e)** Representative images with PAX2/PAX8 immunostaining in *GREB1L*^*WT*^, *GREB1L*^*c.556T>C*^, *GREB1L*^*KO*^, *FGF3*^*WT*^, *FGF3*^*c.493A>G*^, *FGF3*^*KO*^, *PBXIP1*^*WT*^ and *PBXIP1*^*c.1722G>A*^. **(f)** Representative H&E images of *GREB1L*^*WT*^, *GREB1L*^*c.556T>C*^, *GREB1L*^*KO*^, *FGF3*^*WT*^, *FGF3*^*c.493A>G*^, *FGF3*^*KO*^, *PBXIP1*^*WT*^ and *PBXIP1*^*c.1722G>A*^ IEOs on day 25 and day35. *: p<0.05; **: p<0.01; ***: p<0.001.

To determine if candidate variants from patients with cochlear malformations altered the development of the inner ear, IEOs were derived from the monoclonal variant bearing iPSC lines and the isogenic control iPSC lines. Our initial analysis focused on the morphometric characteristics of the IEOs. IEOs were produced using an aggregation approach in 3D suspension culture. All organoids were initiated by seeding 3,500 iPSCs per well of a 96-well low adherence plate. The cross-sectional area of the resulting organoids (variant bearing compared to the WT isogenic control lines) was assessed on day 25 (Fig. 3c and f) and 35 (Fig. 3d and f) after the initiation of IEO differentiation. On day 25, we found that the IEOs derived from all the isogenic controls – *GREB1L*^*WT*^, *FGF3*^*WT*^, and *PBXIP1*^*WT*^ have similar cross-sectional areas. However, all variants of interest show growth restrictions compared to their respective control counterparts -*GREB1L*^*c.556T>C*^ and *GREB1L*^*KO*^ compared to *GREB1L*^*WT*^; *FGF3* ^*c.493A>G*^ and *FGF3*^*KO*^ compared to *FGF3*^*WT*^; *PBXIP1*^*c.1722G>A*^ compared to *PBXIP1*^*WT*^ (Fig. 3c and f). Consistently, the cross-sectional area analysis on day 35 IEOs showed that the variant bearing lines had decreased area compared to their respective isogenic WT controls (Fig. 3d and f). Of note, *FGF3* ^*c.493A>G*^ shows the smallest cross-sectional area of all IEOs (p ≤ 0.001). This is not unexpected since *FGF3* has been shown to work for very early otic structure development (Jeong et al. 2018). In addition to the decrease in cross-sectional area, the HL variant-bearing IEOs had reduced cell confluence in otic vesicles and significantly reduced number of otic vesicles/IEO compared to their isogenic control IEOs (Supplementary Figure S5).

### Inner ear organoids from variant-bearing iPSCs show a lower abundance of otic progenitor markers

PAX2/PAX8 are essential markers for early otic vesicle development and have been previously reported to be associated with IEO development (Li et al. 2024). We have examined PAX2/PAX8 abundance (Figs. 3a, 3b, and 3e) in the otic vesicular area and found that variant-bearing iPSC-derived IEOs showed a lower abundance of early otic progenitor markers (PAX8/PAX2) in *GREB1L*^*c.556T>C*^ (p ≤ 0.01), *FGF3*^*c.493A>G*^ (p ≤ 0.01), *PBXIP1*^*c.1722G>A*^ (p ≤ 0.01), *GREB1L*^*KO*^ (p ≤ 0.01), and *FGF3*^*KO*^ (p ≤ 0.001). *PBXIP1*^*c.1722G>A*^ showed a significant reduction in the abundance of otic progenitor markers (PAX8/PAX2) and overall size reduction, suggesting its pathogenicity in early otic development.

### Inner ear organoids from variant-bearing iPSCs lack hair cell-like populations from mature organoids

**Figure 4:**
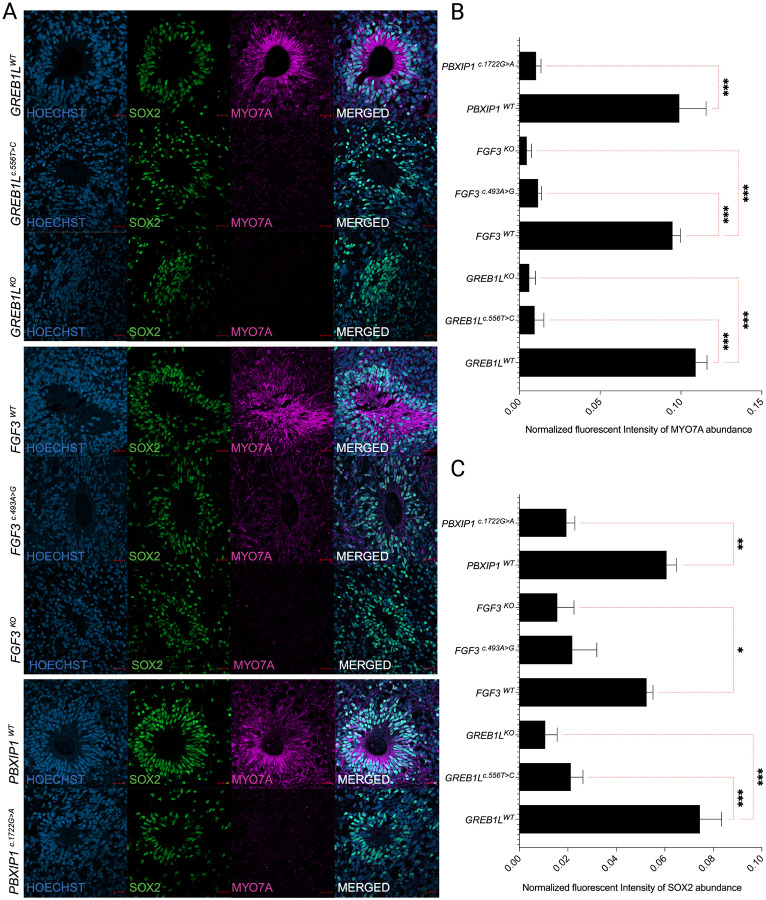
Analysis of the presence of mature hair cell-like populations. **(a)** MYO7A and SOX2 localization in *GREB1L*^*WT*^, *GREB1L*^*c.556T>C*^, *GREB1L*^*KO*^, *FGF3*^*WT*^, *FGF3*^*c.493A>G*^, *FGF3*^*KO*^, *PBXIP1*^*WT*^, and PBXIP1^c.1722G>A^. **(b-d)** Quantification of MYO7A and SOX2 signals in *GREB1L*^*WT*^, *GREB1L*^*c.556T>C*^, *GREB1L*^*KO*^, *FGF3*^*WT*^, *FGF3*^*c.493A>G*^, *FGF3*^*KO*^, *PBXIP1*^*WT*^, and *PBXIP1*^*c.1722G>A*^, respectively (n=3). The results are expressed as Mean ±SEM, and the statistical difference of p ≤ 0.05 was considered significant. The significant differences are marked with (*) whenever comparisons were made between *GREB1L*^*c.556T>C*^, *FGF3*^*c.493A>G*^, *FGF3*^*KO*^, *GREB1L*^*KO*^, *PBXIP1*^*c.1722G>A*^, and their respective controls *GREB1L*^*WT*^, *FGF3*^*WT*^, *PBXIP1*^*WT*^. *: p<0.05; **: p<0.01; ***: p<0.001.

MYO7A and SOX2-positive cells in IEOs are crucial for mimicking the development of the inner ear. These cells define prosensory cell populations that give rise to hair cells and supporting cells essential for hearing and balance functions. In IEOs, MYO7A^+^ and SOX2^+^ hair cell-like cells indicate the development of functional sensory epithelia resembling that found in the inner ear. Organoids derived from *GREB1L*^*c.556T>C*^*, FGF3*^*c.493A>G*^*, PBXIP1*^*c.1722G>A*^*, GREB1L*^*KO*^*, FGF3*^*KO*^, and *PBXIP1*^*c.1722G>A*^ show significantly reduced (p ≤ 0.001) MYO7A^+^ population in comparison to their isogenic controls *GREB1L*^*WT*^*, FGF3*^*WT*^*, and PBXIP1*^*WT*^, respectively (Fig. 4a, 4b). In the inner ear, MYO7A is restricted to the sensory cells of the vestibular and cochlear organs (hair cells). The significant decrease in MYO7A in the HL variant bearing IEOs suggests a defect in the early stages of hair cell development. Additionally, all the organoids derived from variant-bearing iPSC lines show low levels of SOX2, suggesting the presence of a rudimentary supporting cell population (Fig. 4a, 4c).

## Discussion

Here, we present our proof of principle study for the use of human IEOs to rapidly validate uncertain DNA variants detected in patients with cochlear malformations. Moreover, we generated isogenic cell lines from control iPSCs, eliminating the need to obtain patient cells, which can be difficult and time-consuming. From the start of our experiments, the study takes approximately 90 days, making this approach applicable in clinical diagnostics and gene discovery for cochlear malformations.

FGF3 is a small protein (Fig. 1b) serving as a signaling molecule released from the hindbrain during early development. It is implicated in forming prospective sensory tissues along with FGF10. In mice, *Fgf3* is expressed in the otic vesicle during otic placode induction and subsequently at the early stage of inner ear morphogenesis (Hatch et al. 2007; Wilkinson et al. 1988). Models with loss of function variants in FGF3 demonstrated perturbed expression of WNT-induced genes and ultimate patterning defects in the dorsal otocyst. However, the otic genes expressed in ventral otocyst for cochlea development were not critically influenced (Hatch et al. 2007). To the best of our knowledge, our study demonstrates, for the first time, early developmental anomalies of the inner ear in human IEOs and provides functional proof of the role of FGF3 variants detected in affected individuals in driving impairment in early developmental stages of the inner ear.

*GREB1L* encodes a GREB1-like retinoic acid receptor coactivator (Fig. 1b). Several *de novo* and inherited variants in this gene have recently been shown to cause bilateral HL with malformed cochleae (Schrauwen et al. 2018a; Schrauwen et al. 2020a; Schrauwen et al. 2020b). However, no prominent hearing or vestibular defects were observed in zebrafish models for this gene (Schrauwen et al. 2018b). *Greb1l* knockout mice die embryonically, while heterozygous or compound heterozygous mice showed no significant hearing phenotype (www.mousephenotype.org) (De Tomasi et al. 2017). In contrast with previously reported individuals with *GREB1L* variants, our proband has unilateral cochlear aplasia with normal hearing in the other ear. GREB1L is a neural crest regulatory molecule implicated in the embryonic development of many tissues, including the cochlea (Plouhinec et al. 2014). As mutations in other genes involved in neural crest cell migration, such as *PAX3* and *KITL*, have also been shown to cause unilateral HL, the observed clinical phenotype in our proband is not completely surprising (Lee et al. 2023; Zazo et al. 2015). In this study, we show that the mutant and knockout *GREB1L* organoids show decreased expression of otic progenitors and the absence of sensory cells (MYO7A + cells) at the mature stage tested compared to isogenic controls organoids, providing a clarified role of this gene in inner ear development.

*PBXIP1* encodes the PBX homeobox-interacting protein 1 (Fig. 1b), which is involved in cell differentiation through the PI3K/AKT pathway (Manavathi et al. 2012). Previously no evidence has existed that this gene causes inner ear anomalies and deafness in mice or humans. Detection of a loss of function variant, its expression in the cochlea, and role in cell differentiation, made this gene a candidate for inner ear anomalies. The phenotypic and expression data from the organoids establish *PBXIP1*`s role in the development of the inner ear. It is important to point out that the observed abnormalities are identical to those caused by variants in *FGF3* and *GREB1L*, two established genes for cochlear malformations. Additional families with *PBXIP1* variants will secure the establishment of this gene as causing HL.

In summary, we demonstrate the role of *PBXIP1*, *FGF3*, and *GREB1L* in developing otic cells and their differentiation into sensory cells in human organoids. As a study model for HL, organoids may serve as a fast and reliable system for investigating genes involved in cochlear malformations.
